# Deep learning model for individualized trajectory prediction of clinical outcomes in mild cognitive impairment

**DOI:** 10.3389/fnagi.2024.1356745

**Published:** 2024-05-15

**Authors:** Wonsik Jung, Si Eun Kim, Jun Pyo Kim, Hyemin Jang, Chae Jung Park, Hee Jin Kim, Duk L. Na, Sang Won Seo, Heung-Il Suk

**Affiliations:** ^1^Department of Brain and Cognitive Engineering, Korea University, Seoul, Republic of Korea; ^2^Department of Neurology, Samsung Medical Center, Sungkyunkwan University School of Medicine, Seoul, Republic of Korea; ^3^Department of Neurology, Inje University College of Medicine, Haeundae Paik Hospital, Busan, Republic of Korea; ^4^Neuroscience Center, Seoul, Republic of Korea; ^5^Alzheimer’s Disease Convergence Research Center, Samsung Medical Center, Seoul, Republic of Korea; ^6^Department of Neurology, Seoul National University Hospital, Seoul National University College of Medicine, Seoul, Republic of Korea; ^7^National Cancer Center Research Institute, Goyang, Republic of Korea; ^8^Department of Health Sciences and Technology, SAIHST, Sungkyunkwan University, Seoul, Republic of Korea; ^9^Center for Clinical Epidemiology, Samsung Medical Center, Seoul, Republic of Korea; ^10^Clinical Research Design and Evaluation, SAIHST, Sungkyunkwan University, Seoul, Republic of Korea; ^11^Department of Artificial Intelligence, Korea University, Seoul, Republic of Korea

**Keywords:** deep learning, mild cognitive impairment, predictive model, cognitive decline, Alzheimer’s disease, prognosis, magnetic resonance imaging, missing value imputation

## Abstract

**Objectives:**

Accurately predicting when patients with mild cognitive impairment (MCI) will progress to dementia is a formidable challenge. This work aims to develop a predictive deep learning model to accurately predict future cognitive decline and magnetic resonance imaging (MRI) marker changes over time at the individual level for patients with MCI.

**Methods:**

We recruited 657 amnestic patients with MCI from the Samsung Medical Center who underwent cognitive tests, brain MRI scans, and amyloid-β (Aβ) positron emission tomography (PET) scans. We devised a novel deep learning architecture by leveraging an attention mechanism in a recurrent neural network. We trained a predictive model by inputting age, gender, education, apolipoprotein E genotype, neuropsychological test scores, and brain MRI and amyloid PET features. Cognitive outcomes and MRI features of an MCI subject were predicted using the proposed network.

**Results:**

The proposed predictive model demonstrated good prediction performance (AUC = 0.814 ± 0.035) in five-fold cross-validation, along with reliable prediction in cognitive decline and MRI markers over time. Faster cognitive decline and brain atrophy in larger regions were forecasted in patients with Aβ (+) than with Aβ (−).

**Conclusion:**

The proposed method provides effective and accurate means for predicting the progression of individuals within a specific period. This model could assist clinicians in identifying subjects at a higher risk of rapid cognitive decline by predicting future cognitive decline and MRI marker changes over time for patients with MCI. Future studies should validate and refine the proposed predictive model further to improve clinical decision-making.

## Introduction

Amnestic mild cognitive impairment (aMCI) is considered the preceding phase of dementia in Alzheimer’s disease (AD) (Zhu et al., 2013). Approximately 10–15% of patients with aMCI develop AD dementia per year, with an average conversion rate of 60% after 5 years (Farias et al., 2009). However, progression is variable among individuals with aMCI. Due to the heterogeneity in its etiology, different rates of cognitive decline are observed in patients with aMCI. While some patients exhibit rapid conversion to AD dementia, others remain stable or may even return to normal cognitive functioning (Busse et al., 2006). The deposition of amyloid-β (Aβ), a well-known pathological hallmark of AD, is a critical predictor of conversion to AD in aMCI patients. Indeed, it has been found that only 40 to 60% of patients diagnosed with aMCI exhibit Aβ positivity (Landau et al., 2012; Roberts et al., 2018), and the conversion rate for Aβ (+) patients with aMCI is 4 to 9 times higher than that of Aβ negative, Aβ (−) (Okello et al., 2009; Doraiswamy et al., 2014).

Recent treatment strategies for AD focus on the predementia stage encompassing MCI and aim at slowing cognitive deterioration. Identifying the timing when individuals with MCI would benefit from treatment is essential. Hence, recent studies have shifted its emphasis from the follow-up to predicting the time of progression in individuals with MCI (Li et al., 2013, 2018). Promising results have been achieved using clinical and imaging-based parameters at the baseline and employing their longitudinal change pattern to forecast of the progression of MCI to AD dementia in individuals.

Further, advances in technology have made deep learning capable of creating new prediction models. Previous studies have shown the newly developed deep learning methods to detect MCI and AD. These studies have demonstrated the ability to evaluate features of abnormal brain connections or identify discriminative brain regions of AD (Pan et al., 2021; Zuo et al., 2021, 2024). In contrast to conventional machine learning models, deep learning techniques have emerged as powerful tools capable of effectively analyzing high-dimensional data and capturing the intricate and nonlinear relationships between its features (Jung et al., 2021). Building on our previous work (Jung et al., 2021), we demonstrated the feasibility of devising a deep recurrent network to address the four interrelated problems of missing value imputation, phenotypic measurement forecasting, cognitive score trajectory estimation, and clinical status prediction using longitudinal imaging biomarkers (Jung et al., 2021). The proposed method is superior to the existing competing methods for a variety of quantitative metrics (Jung et al., 2021). To our knowledge, no existing research has reported the application of machine or deep learning models for predicting the future outcomes of patients with MCI regarding Aβ (+) and Aβ (−) in the context of AD progression.

The present study aims to extend a predictive model using multimodal biomarkers to forecast cognitive decline and magnetic resonance imaging (MRI) markers in the Aβ (+) and Aβ (−) MCI populations. We hypothesize that clinical, neuropsychological, and neuroimaging features of the characteristics of aMCI exhibit associations with cognitive decline and MRI markers, and a combination of these features could enable accurate individual-level predictions of cognitive decline and MRI markers. Under this assumption, we aim to enhance our understanding of the relationship between these features in aMCI and their respective implications for cognitive decline and MRI markers.

## Dataset and preprocessing

### Study participants

We recruited 657 patients with aMCI who underwent three-dimensional (3D) MRI and Aβ positron emission tomography (PET) imaging (^18^F-florbetaben (FBB) PET or ^18^F-flutemetamol (FMM)) imaging between February 2015 and June 2021 at Samsung Medical Center (SMC). All participants diagnosed with MCI fulfilled the following criteria (Albert et al., 2011): (1) Participants or their caregivers must report subjective cognitive complaints. (2) Participants must exhibit objective cognitive impairment in any cognitive domain, with scores falling below −1.0 standard deviations of age- and education-matched norms in memory and − 1.5 standard deviations in other cognitive domains. (3) Participants must not have significant impairment in activities of daily living. (4) Participants must not have dementia. We excluded laboratory-confirmed secondary causes of cognitive deficits, including thyroid, renal, and hepatic function tests, vitamin B12, and syphilis serology. Individuals who had structural abnormalities on their brain MRI, such as territorial infarctions, intracranial hemorrhages, brain tumors, hydrocephalus, or significant white matter hyperintensities as determined by the modified Fazekas ischemia scale (Noh et al., 2014), were also excluded.

This study was approved by the Institutional Review Board of SMC (IRB No: 2018–10-120). Written informed consent was obtained from the patients and their caregivers.

### Neuropsychological tests

All participants underwent the Seoul Neuropsychological Screening Battery second edition (SNSB-II) (Kang et al., 2003, 2019). All of the included tests in SNSB-II have been internationally used for several decades in clinical practice (Wechsler, 1955; Folstein et al., 1975; Golden, 1978; Benton et al., 1983; Kaplan et al., 1983; Morris, 1993; Meyers and Meyers, 1995; Kang et al., 1997, 2003, 2012; Benedict et al., 1998; O'Bryant et al., 2008; Ryu and Yang, 2023). The items used in the tests were altered due to the linguistic and cultural differences between Korean and English speakers (Kim and Na, 1999; Kang et al., 2003, 2012; Ryu and Yang, 2023). We used tests that provided numerical scores, such as the Digit Span Forward (DSF), the Korean version of the Boston Naming (K-BNT), Rey complex figure test (RCFT) (copying and delayed recall), Seoul verbal learning test (SVLT) (delayed recall), semantic controlled oral word association test (COWAT), Stroop Test (color reading), Korean-Mini Mental State Examination (K-MMSE), and Clinical Dementia Rating-Sum of Boxes (CDR-SB). In the analysis, the results with numerically continuous values were used.

The participants’ attention and working memory were assessed using the DSF, and naming ability was evaluated using the K-BNT score. Verbal memory and visual memory were measured using the SVLT (delayed recall) and RCFT (delayed recall) scores, respectively. The visuospatial function was assessed using the RCFT copying test, and the frontal executive function was measured using the semantic COWAT and Stroop test. The global cognition was evaluated with K-MMSE and CDR-SB.

### MRI data processing for cortical thickness measurements

All subjects received 3D T1 turbo field echo images and 3D fluid-attenuated inversion recovery at SMC with a 3.0 T MRI scanner (Philips 3.0 T Achieva; Philips Healthcare, Andover, MA, USA), as previously described. The CIVET anatomical pipeline (v. 2.1.0) was used to process the images (Zijdenbos et al., 2002). The MRI images of the native subjects were aligned with the MNI-152 template using a linear transformation method (Collins et al., 1994). Additionally, the images were adjusted for variations in intensity using the N3 algorithm (Sled et al., 1998). The images that were registered and corrected were segmented into distinct regions, including white matter, gray matter, cerebrospinal fluid, and background. Furthermore, the marching-cubes approach was employed to automatically extract the inner and outer cortex surfaces. This allowed for the calculation of cortical thickness, which is defined as the Euclidean distance between the connected vertices of the inner and outer surfaces (Lerch and Evans, 2005).

Intracranial volume (ICV) was calculated by measuring the total volumes of the voxels within the skull-stripped brain mask. After obtaining cortical surface models using MRI volumes that were converted into stereotaxic space, we evaluated the cortical thickness in the original space by using an inverse transformation matrix to rebuild the cortical surface in the original space (Im et al., 2006).

We utilized a surface-based 2D registration technique employing a sphere-to-sphere warping algorithm. Furthermore, we spatially standardized the cortical thickness values to facilitate a comparison between the thickness obtained from the registration algorithm and an unbiased iterative group template with improved anatomical detail (Lyttelton et al., 2007). This transformation allowed us to align the thickness information for the vertices with the unbiased iterative group template. The technique of surface-based diffusion smoothing was employed to blur each cortical thickness map, with a full width at half maximum of 20 mm. This process was done to enhance the signal-to-noise ratio and statistical power of the data, as described by previous studies (Chung et al., 2003; Im et al., 2006). In order to quantify the hippocampal volume (HV), we employed an automated method for segmenting the hippocampus. This method utilized a graph cut algorithm in conjunction with atlas-based segmentation and a morphological opening technique, as detailed in an earlier study.

### Amyloid PET imaging acquisition, analysis, and Centiloid values

All participants underwent either FBB or FMM PET scans at SMC using a Discovery STe PET/CT scanner (GE Medical Systems, Milwaukee, WI, USA). The scans were performed in 3D mode, examining 47 slices of 3.3 mm thickness that covered the entire brain (Jang et al., 2019). The CT pictures were obtained using a 16-slice helical CT scanner with a section width of 3.75 mm. The scanner was set to 140 keV and 80 mA for attenuation correction. Following the guidelines provided by the makers of the ligands, a dynamic emission PET scan lasting 20 min was conducted. This scan consisted of four frames, each lasting 5 min. The scan was performed 90 min after injecting an average dose of 311.5 MBq of FBB or 185 MBq of FMM. The 3D PET scans were reconstructed using the ordered-subset expectation–maximization algorithm with a voxel size of 2.00 × 2.00 × 3.27 mm. The reconstruction was done in a 128 × 128 × 48 matrix. The algorithm parameters used were FBB iterations = 4 and subsets = 20 for the ordered-subset expectation–maximization algorithm, and FMM iterations = 4 and subsets = 20 for the same algorithm. The PET images were aligned with individual 3D-T1 weighted MRI scans that were standardized to the T1-weighted MNI-152 template using statistical parametric mapping (SPM) 8. The quantification of Aβ uptakes was performed using BeauBrain Morph, a software developed by BeauBrain Healthcare Co., Ltd. This software utilizes fully-automated image processing to measure Aβ uptakes on PET scans. In the previous study, to improve the prediction of prognosis and early detection, we developed an MRI-based regional modified Centiloid (rdcCL) method that harmonizes the overall and regional Aβ uptake among Aβ ligands (Klunk et al., 2015). More details of the analysis method are found in the original Centiloid project paper and previous paper (Kim et al., 2022). The MRI and PET images underwent spatial normalization using the transformation parameters obtained from SPM8. The whole cerebellar (WC) mask for the reference region was obtained from the Global Alzheimer’s Association Interactive Network website.^1^ We created a WC mask for rdc-SUVR to calculate global and regional Centiloid using FBB and FMM PET images of Aβ patients. The six regional Volume of interests (VOIs) were named the frontal, lateral temporal, occipital, parietal, posterior cingulate, and striatal areas. rdc-SUVR values were calculated using the global regional VOIs. We divided the groups into two groups using the K-means clustering algorithm, and the cut-off was obtained by using the Centiloid values of patients in the lower group of these two groups. We defined Aβ positivity based on the cutoff value of the FBB or FMM PET global rdcCL, which was previously computed as 27.08 (Jang et al., 2021). This cutoff value is similar to the cutoff of 30 CL which was reported by previously studies (Salvadó et al., 2019; Milà-Alomà et al., 2021, 2022).

## Proposed method

**Figure 1:**
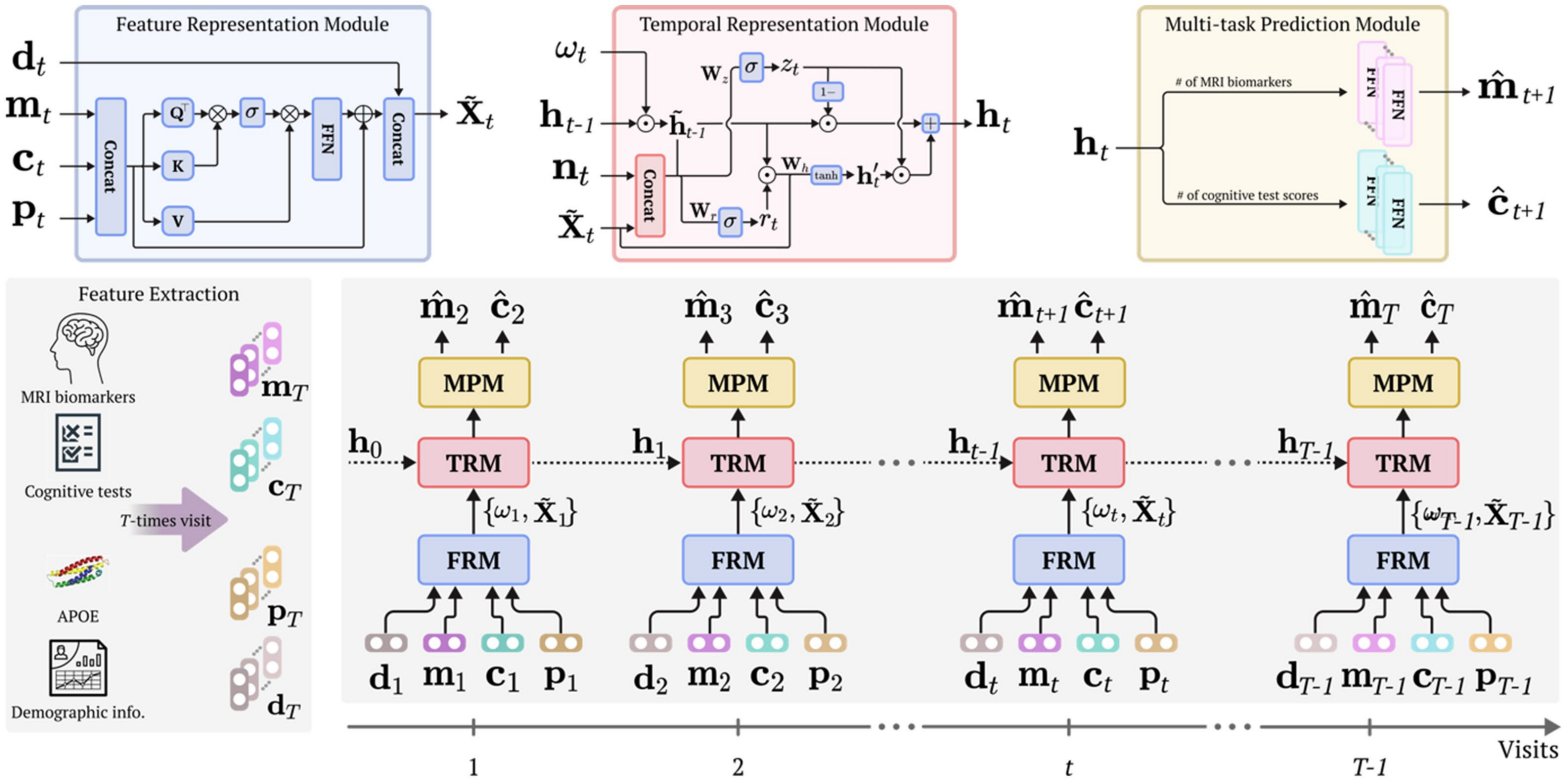
Overall framework. Our proposed framework comprises a feature representation module (FRM) to learn enriched feature representation and to fuse multimodal input features using a self-attention mechanism; a temporal representation module (TRM) dedicated to accounting for the characteristics of temporal dynamics; and a multi-task prediction module (MPM) aimed at predicting the MRI biomarkers and cognitive test score for subsequent time visits. Before our proposed framework is trained, the data extraction procedure is initially conducted, utilizing MRI, cognitive tests, *APOE* genotype, and demographic data. Henceforth, each module considers multivariate and temporal traits, thereby using various input features from the current visit to predict the MRI biomarkers and cognitive test scores for the subsequent visit. FRM, feature representation module; TRM, temporal representation module; MPM, multi-task prediction module; APOE, apolipoprotein E.

This work proposes a simple but efficient framework for modeling personalized prognostic trajectories in participants with aMCI. Specifically, the proposed framework consists of three modules: a feature representation module (FRM), temporal representation module (TRM), and multi-task prediction module (MPM) as illustrated in Figure 1. In the FRM, we employ a self-attention mechanism (Vaswani et al., 2017) to fuse diverse clinical information (e.g., MRI markers, cognitive test scores, and the presence of the apolipoprotein E (*APOE*) carrier) into representative features. This module captures complex relationships of the input data and provides a comprehensive representation. The representative features were input into the TRM. Through this process, the proposed framework embeds and captures the underlying temporal characteristics inherent in the longitudinal data. The temporally embedded features are then fed into the MRM to simultaneously predict the cortical thickness of MRI markers and cognitive test scores for the next time sequence. By integrating these three modules, our proposed framework facilitates modeling individualized prognostic trajectories in participants diagnosed with aMCI.

### Notation

In this work, we express matrices with boldface uppercase letters, vectors with boldface lowercase letters, and scalars with italic letters. For a given matrix C , we define its $\left(i,j\right)$ th element and the i -th column as $C\left(i,j\right)$ and $c_{i}$ , respectively. Thus, the longitudinal neuroimaging dataset is characterized by a multivariate time series consisting of B variables across T time sequences from N number of samples as ${\left\{X^{\left(n\right)},Y^{\left(n\right)}\right\}}_{n=1}^{N}\mathrm{where}\;X^{\left(n\right)}=\left[x_{1}^{\left(n\right)},\cdots ,x_{t}^{\left(n\right)},\cdots ,x_{T}^{\left(n\right)}\right]\in {\mathbb{R}}^{\left(B\times T\right)}\text{,}$ $Y^{\left(n\right)}=\left[y_{1}^{\left(n\right)},\cdots ,y_{t}^{\left(n\right)},\cdots ,y_{T}^{\left(n\right)}\right]\in {\left\{0,1\right\}}^{L\times T}\text{,}$ and L represents the of classes, number of classes, such as Aβ (+) and Aβ (−) status. Furthermore, each data point integrates demographic information (i.e., age, gender, and education) pertaining to the n -th sample from the first visit (i.e., baseline). To use them as input features, demographic information is duplicated across T time sequences, with the exception of age. For simplicity, the superscript *n* is omitted. For clarity, the input features are defined as $x_{t}=\left\{m_{t},c_{t},p_{t},d_{t}\right\}$ , where $m_{t}$ denotes the cortical thickness features, $c_{t}$ represents the cognitive test scores, $p_{t}$ indicates *APOE* features, and $d_{t}=\left\{d_{t}^{\mathrm{age}},d_{t}^{edu},d_{t}^{gen}\right\}$ represents the demographic information, including age, education, and gender.

### Missing value imputation

Given the scarcity of longitudinal data $\left\{X,Y\right\}$ , we tackle the problem of missing values by applying an imputation technique. To achieve this, we employ a mask vector alongside a time interval between consecutive data observations. These variables-the mask vector and time interval-are instrumental in informing to the model about the presence or absence of input features and labels. Consequently, this facilitates the model’s capacity to adeptly manage missing values, thereby recuperating essential information both for the imputation of missing values and for the latent feature representation. To incorporate this information, we employed a time delay matrix, indicating the time interval of observation. Harnessing these characteristics, we embraced a data-driven imputation strategy, which proficiently utilizes both temporal and multivariate relationships to impute missing observations. A detailed explanation is provided in our earlier work (Jung et al., 2021).

### Feature representation module

The proposed framework encompasses an feature representation module (FRM) designed to identify and understand the relationships among multimodal input data, thereby learning an enriched representation of clinical information. In other words, this module leverages a self-attention mechanism, thereby identifying feature-specific relationships and acquiring intricate and comprehensive clinical information.

To adhere to the aforementioned assumption, at time t, we initially concatenate the input features that include MRI markers, cognitive test scores, and the presence of *APOE* carriers as follows:


$${\tilde{x}}_{t}=m_{t}⊚c_{t}⊚p_{t}\left(\in {\mathbb{R}}^{\left(B^{\prime }\times 1\right)}\right)\text{,}$$

where ⊚ represents a concatenation operator. Subsequently, these concatenated features are transformed into three distinct representations through the utilization of three feed-forward networks (FFNs): the query $\left(Q\right)$ , key $\left(K\right)$ , and value $\left(V\right)$ :


$$Q={\tilde{x}}_{t}W_{q},K={\tilde{x}}_{t}W_{k},V={\tilde{x}}_{t}W_{v}\text{,}$$

where $W_{q}\in {\mathbb{R}}^{\left(B^{\prime }\times B^{\prime }\right)},W_{k}\in {\mathbb{R}}^{\left(B^{\prime }\times B^{\prime }\right)}\text{,}$ and $W_{v}\in {\mathbb{R}}^{\left(B^{\prime }\times B^{\prime }\right)}$ are learnable weight matrices. Following this, the similarity between each query in the Q representation and each key in K representation is computed using the elementwise product. A standard self-attention calculation (Vaswani et al., 2017) is then executed as follows:


$$\mathrm{Attention}\left(Q,K\right)=\sigma \left(\frac{QK^{⊺}}{\sqrt{d_{k}}}\right)\text{,}$$

where $d_{k}$ representing the feature dimension of K and σ denotes a softmax function. After performing the self-attention operation, the outcome is multiplied by the value features. FFN is then applied to further refine the transformed features. These refined features are added back to the original concatenated features (i.e., attentive feature vectors) and concatenated with the demographic features as outlined in Figure 1 (FRM).

### Temporal representation module

Following the combining of attentive feature vectors with demographic features ( ${\tilde{x}}_{t}\in {\mathbb{R}}^{\left(B^{\prime }\times 1\right)}$ ), we employed a gated recurrent unit (GRU) network (Chung et al., 2014) to capture the temporal representation. We introduce a novel computational strategy via a modified GRU cell incorporating a temporal decaying factor $\omega_{t}\in {\mathbb{R}}^{\left(H\times 1\right)}$ (where H denotes the number of hidden units) and a mask vector $n_{t}\in {\mathbb{R}}^{\left(B^{\prime }\times 1\right)}$ to delineate whether each observation was directly observed or imputed. The hidden state $h_{t-1}\in {\mathbb{R}}^{\left(H\times 1\right)}$ of the recurrent network embodies temporal context information up to the previous time $\left(t-1\right)$ ; nonetheless, it is crucial to deliberate on the manner of amalgamating this temporal data with the current observation, particularly concerning the term of recent true observation. To address this, we leverage a temporal decaying factor that judiciously apportions the impact of past observations, thereby effectively embedding them with the current observation as follows: ${\tilde{h}}_{t-1}=h_{t-1}⊙\omega_{t}$ , where ⊙ denotes an elementwise multiplication operator. In doing so, the TRM captures temporal dependencies and adjusts its representation accordingly. Moreover, the inclusion of the mask vector into the TRM informs the model about the imputed values. Through the using of temporal patterns and mask vectors, our proposed framework adeptly discerns imputation patterns, thereby improving its representational capability.

Before demonstrating the modified gating operation of the GRU cell, we introduce the role of its two gates (i.e., reset gate and update gate). These gates control the flow of information. Specifically, the reset gate determines how much of the previous hidden state should be forgotten or reset before considering the current input, while the update gate controls how much of the new hidden state should be updated with the current input. By dynamically adjusting the reset and update gates, the GRU cell can selectively remember or forget information from the past, allowing it to capture long-term dependencies in sequential data. The operation of TRM within the proposed framework is as follows:


$$r_{t}=\sigma \left(W_{r}⊙\left[{\tilde{h}}_{t-1},{\tilde{x}}_{t}⊚n_{t}\right]+b_{r}\right)\text{,}$$

$$z_{t}=\sigma \left(W_{z}⊙\left[{\tilde{h}}_{t-1},{\tilde{x}}_{t}⊚n_{t}\right]+b_{z}\right)\text{,}$$

$$h\prime_{t}=\tanh\left(W_{h}⊙\left[r_{t}⊙{\tilde{h}}_{t-1},{\tilde{x}}_{t}\right]\right)\text{,}$$

$$h_{t}=\left(1-z_{t}\right)⊙{\tilde{h}}_{t-1}+z_{t}⊙h\prime_{t}\text{,}$$

where $\left\{W_{z},W_{r},W_{h},b_{z},b_{r}\right\}$ represent learnable parameters of the modified GRU cell. The outputs of the update and reset gates are denoted by $r_{t}$ and $z_{t}$ , respectively, while $h\prime_{t}$ represents the candidate’s hidden state, and σ denotes the sigmoid function. Each weight vector (i.e., $W_{z},W_{r},W_{h}$ ) comprises two internal vectors: $W_{\left(\mathrm{gate}\right)i}\in {\mathbb{R}}^{\left(H\times 2B^{\prime }\right)}$ and $W_{\left(\mathrm{gate}\right)h}\in {\mathbb{R}}^{\left(H\times H\right)}$ , where $\left(\mathrm{gate}\right)\in \left\{z,r,h\right\}$ . Likewise, each bias vector has the same conditions, where the subscript i represents the connection between the weight vector and an input ${\tilde{x}}_{t}⊚n_{t}$ , and the subscript h represents the connection between the weight vector and temporal context information ${\tilde{h}}_{t-1}$ . The computation process is summarized as follows:


$$r_{t}=\sigma \left(W_{r}⊙\left[{\tilde{h}}_{t-1},{\tilde{x}}_{t}⊚n_{t}\right]+b_{r}\right)\text{,}$$

$$r_{t}=\sigma \left(W_{ri}⊙\left({\tilde{x}}_{t}⊚n_{t}\right)+b_{ri}+W_{rh}⊙{\tilde{h}}_{t-1}+b_{rh}\right)\text{.}$$

### Multi-task prediction module

Based on the hidden representation $h_{t}$ from the TRM, the multi-task prediction module (MPM) generates two predictions for the next time point: the predicted MRI biomarker ${\hat{m}}_{t+1}$ and the predicted cognitive test scores ${\hat{c}}_{t+1}$ . For each outcome, we employ simple linear regression models, defined as follows:


$${\hat{m}}_{t+1}=W_{m}h_{t}+b_{m}\text{,}$$

$${\hat{c}}_{t+1}=W_{c}h_{t}+b_{c}\text{,}$$

where $W_{m},W_{c},b_{m}$ , and $b_{c}$ denote the learnable parameters of the linear regression models. The MPM is connected with FRM and TRM, allowing for the joint optimization of the parameters across all three modules.

### Optimization and algorithm

We formulated a composite objective function to simultaneously train the proposed framework. Specifically, for the prediction of the MRI biomarker (i.e., cortical thickness), denoted as $L_{m}$ , we computed the mean squared error (MSE) between the model output ${\hat{m}}_{t+1}$ from the MPM and the corresponding true observations $m_{t+1}$ :


$$L_{m}=\overset{T-1}{\underset{t=1}{\sum }}{\left(m_{t+1}-{\hat{m}}_{t+1}\right)}^{2}\text{.}$$

Similarly, for the prediction of cognitive test scores, denoted as $L_{c}$ , we measured the congruity between the model output ${\hat{c}}_{t+1}$ from the MPM and the actual cognitive test score $c_{t+1}$ :


$$L_{c}=\overset{T-1}{\underset{t=1}{\sum }}{\left(c_{t+1}-{\hat{c}}_{t+1}\right)}^{2}\text{.}$$

Finally, the overall loss function $L_{\mathrm{total}}$ was defined as a weighted combination of the MRI biomarker loss $L_{m}$ and the cognitive test score loss $L_{c}$ :


$$L_{\mathrm{total}}=\alpha L_{m}+\gamma L_{c}\text{,}$$

where α and γ are hyperparameters to balance the influence of the respective losses. The optimization of this objective function enables the training of all learnable parameters in the proposed framework using the stochastic gradient descent method in an end-to-end manner. We performed the objective functions in various settings, including the MSE and mean absolute error (MAE). However, based on the experimental results, we selected MSE as the preferred metric.

## Experiments and results

### Participant characteristics and demographics

A total of 657 aMCI participants were included in the study. Table 1 presents the demographic characteristics of the participants. The mean age of the study participants was 71.5 ± 8.2 years, and 56.0% (*n =* 368) were females. Among participants, 312 (41.5%) were Aβ (−), and 345 (52.5%) were Aβ (+). No statistical differences were found in the mean age (*p* = 0.933), the proportion of females (*p* = 0.329), or years of education (*p* = 0.902) between the participants with Aβ (−) and Aβ (+) aMCI. Participants with Aβ (+) aMCI had a higher frequency of the *APOE* ε4 genotype (*p* < 0.001) than participants with Aβ (−) aMCI. Furthermore, statistically significant disparities were observed in most cognitive scores, with the exceptions of DSF, COWAT, and K-BNT. Among these assessments, CDR-SB and K-MMSE are known as pivotal tools for assessing the severity of dementia and cognitive impairment (Morris, 1993; Kang et al., 1997; O'Bryant et al., 2008). Based on these cognitive scores, it was observed that participants with Aβ (+) aMCI exhibited notably higher levels of cognitive impairment severity compared to those with Aβ (−) aMCI. Consequently, a higher score on the CDR-SB indicates greater dementia severity, while a lower score on the K-MMSE signifies more severe cognitive deficits. Based on the outcomes presented in Table 1, we validated the hypothesis that categorizing aMCI patients into Aβ (+) and Aβ (−) groups through quantitative criteria is plausible and provides a theoretical basis for developing treatment strategies for dementia and cognitive impairment. Furthermore, this approach reaffirmed the utility of CDR-SB and K-MMSE in assessing cognitive impairment.

**Table 1:** Baseline demographic characteristics of Aβ (−) and Aβ (+) aMCI.

Variables	Total (*n* = 657)	Aβ (−) aMCI (*n* = 312)	Aβ (+) aMCI (*n* = 345)
Baseline age, years^a^ (−)	71.5 ± 8.2	71.0 ± 8.3	72.0 ± 8.2
Education, years^a^ (−)	12.1 ± 4.5	12.4 ± 4.5	11.8 ± 4.4
Female, *N* (%) (−)	368 (56.0)	158 (50.6)	210 (60.9)
*APOE* ε4 carrier, *N* (%) (*p* < 0.001)	276 (42.0)	53 (17.0)	223 (64.6)
CDR-SB^a^ (*p* < 0.001)	1.6 ± 1.1	1.3 ± 0.9	1.9 ± 1.2
K-MMSE^a^ (*p* < 0.001)	25.9 ± 3.0	26.8 ± 2.4	25.1 ± 3.3
DSF^a^ (−)	5.9 ± 1.4	5.9 ± 1.4	5.9 ± 1.4
K-BNT^a^ (−)	42.7 ± 9.3	43.2 ± 9.3	42.3 ± 9.4
RCFT-Copy^a^ (*p* < 0.01)	29.9 ± 6.8	30.8 ± 5.7	29.1 ± 7.5
SVLT-Delayed recall^a^ (*p* < 0.001)	2.5 ± 2.5	3.4 ± 2.5	1.6 ± 2.1
RCFT-Delayed recall^a^ (*p* < 0.001)	6.8 ± 5.6	9.0 ± 6.1	4.7 ± 4.3
COWAT^a^ (−)	22.5 ± 11.1	22.2 ± 10.0	22.7 ± 12.0
Stroop color reading^a^ (*p* < 0.001)	67.6 ± 27.9	72.5 ± 26.4	63.2 ± 28.4
Baseline Aβ uptake (dcCL)^a^ (*p* < 0.001)	49.3 ± 49.0	7.5 ± 18.8	90.9 ± 31.1
Follow-up duration, years^a^	2.1 ± 1.5	1.9 ± 1.3	2.3 ± 1.7

^a^Values are the mean and standard deviation. Aβ, Amyloid-β; aMCI, amnestic mild cognitive impairment; N, number; APOE ε4, apolipoprotein E ε4 allele; CDR-SB, Clinical Dementia Rating Scale Sum of Boxes; COWAT, controlled oral word association test; dcCL, direct comparison centiloid unit; DSF, digital span forward; K-BNT, Korean version of the Boston Naming Test; RCFT, Rey complex figure test; SVLT, Seoul verbal learning test; K-MMSE, Korean-Mini Mental State Examination.

### Experimental settings

We validated the effectiveness of the proposed framework, which employs a systematic data-driven approach for multitask learning to model personalized prognostic trajectories in patients with MCI. Specifically, the proposed framework focuses on forecasting MRI markers and cognitive test scores, which are crucial indicators in assessing disease progression. We made predictions for each time point, with a one-year interval covering four consecutive time sequences, corresponding to forecasting the outcomes for the subsequent 3 years following the baseline. In the prediction process, we applied all available historical data, including observed measurements and imputed values for any missing or unobserved data. By incorporating this comprehensive information, the framework provides accurate and reliable predictions for each time sequence, enabling a thorough assessment of the future progression of the disease.

To ensure the reliability of the experimental results, we conducted a rigorous evaluation using five-fold cross-validation with five repetitions. In addition, the dataset was randomly partitioned into three mutually exclusive subsets: training, validation, and testing. Specifically, we randomly sampled 10% of the subjects from each class as the validation set, whereas another 10% were selected as the testing set from the baseline time point. This approach allowed the framework to validate the performance of the proposed model on unseen data and mitigate the influence of any specific subset of subjects. Regarding training settings, we performed a grid search for hyperparameter selection, including $5\times \left\{10^{-5},10^{-4},10^{-3},10^{-2}\right\}$ for the learning rate, $\left\{1,2,3\right\}$ for the number of hidden layers, $\left\{16,32,48,64,80,96\right\}$ for the size of the hidden units, and $\left\{10^{-6},10^{-5},10^{-4},10^{-3}\right\}$ for the $l_{2}$ -regularization.

Subsequently, we leveraged an early stopping strategy to identify the optimal hyperparameters by minimizing the MSE on the validation set. In terms of the proposed framework, we used FFNs in the FRM and MPM. In particular, the FRM consisted of 17 input nodes ( $B^{\prime }$ ) and 17 output nodes for the query, key, value, and gating layer. In MPM, we employed 21 input nodes ( B ) and six (*number of MRI markers*) and nine (*number of cognitive test scores*) output nodes for regression layers. Furthermore, we exploited a GRU cell in the TRM, consisting of 21 input nodes and 48 hidden states. To ensure a balanced optimization process, we introduced loss balance control by setting α and γ to 0.75 and 1.0, respectively. We implemented our proposed framework with PyTorch, and we trained them with Titan RTX GPU on Ubuntu 18.04.

For the quantitative evaluation, we used the MAE, mean absolute percentage error (MAPE), and coefficient of determination ( $R^{2}$ ) for the MRI biomarker prediction and cognitive test scores prediction tasks. We also conducted statistical significance tests, including Pearson’s correlation coefficient for downstream tasks. For comparison with the other methods, we utilized the paired, two-sided Wilcoxon signed-rank test (Wilcoxon, 1992).

We compared our proposed method against the following approaches, which address tasks related to imputing missing values and forecasting:

- Mean imputation combined with GRU (GRU-M): missing observations were imputed using the mean values of the respective variables from the training data. Subsequently, a GRU cell was employed for forecasting tasks, such as cognitive test scores and MRI markers.
- Multi-directional recurrent neural network (M-RNN) (Yoon et al., 2018): This model, a variant of the traditional RNN, is designed to process data across multiple directions. Specifically, M-RNN leverages the concept of a bidirectional RNN to interpolate missing information within individual data streams, allowing it to analyze data in a more interconnected stream than in isolation. This makes it valuable for handling complex, multi-stream datasets where understanding the context and correlation between data points is key to accurate forecasting and imputations. However, M-RNN does not consider correlations among features.
- Self-Attention-based Imputation for Time Series (SAITS) (Du et al., 2023): This model is trained with a joint optimization approach that utilizes two diagonal-masked self-attention blocks (DMSA) to effectively capture both the temporal dependencies and feature correlations between time steps, thereby improving imputation accuracy and training speed. In addition, a weighted-combination block dynamically assigns weights to the representations learned from two DMSA blocks, guided by attention weights and missingness information, to further refine imputation precision.

For all comparative methods, the range of initial hyperparameters was set based on their original papers, and the optimal hyperparameters were selected based on the results of the validation set. All of the experiments were conducted using the same experimental settings as that of the proposed method.

### Prediction of cognitive test scores

Table 2 summarizes the prediction errors for the longitudinal changes in neuropsychological test scores. Between the proposed framework and comparative methods. Namely, we evaluated the prediction errors using MAE, MAPE, and R^2^ metrics. Note that the values for the highest performance are highlighted in bold, while the second-highest performance is denoted with an underline. First, it is noteworthy to highlight that our proposed method outperformed all the competing methods under our consideration, achieving the lowest MAE, MAPE, and *R*^2^ scores, with a statistical significance of p<0.05 for most of the competing methods. Overall, M-RNN and SAITS demonstrated better performance than GRU-M across all cognitive test scores in terms of MAPE and R^2^ metrics. However, in terms of MAE, M-RNN showed lower performance than GRU-M specifically in the RCFT-Copy score, though it still outperformed GRU-M in terms of MAPE and R^2^. This underscores the importance of utilizing a variety of evaluation metrics rather than relying on a single one to ensure a fair comparison. Additionally, we observed that among M-RNN and SAITS, the SAITS approach generally yielded better performance than M-RNN across all metrics, except for the COWAT score.

**Table 2:** Performance predicting normalized cognitive test scores for MAE, MAPE, and *R*^2^ (^†^*p*<0.05).

Features	Methods	MAE↓	MAPE↓	R^2^↑
DSF	GRU-M	0.109 ± 0.012^†^	0.176 ± 0.010^†^	0.293 ± 0.030^†^
	M-RNN	0.109 ± 0.013^†^	0.171 ± 0.023^†^	0.354 ± 0.066^†^
	SAITS	0.096 ± 0.013^†^	0.157 ± 0.016^†^	0.401 ± 0.071^†^
	**Ours**	**0.089 ± 0.016**	**0.140 ± 0.021**	**0.524 ± 0.101**
K-BNT	GRU-M	0.112 ± 0.005^†^	0.241 ± 0.057^†^	0.345 ± 0.099^†^
	M-RNN	0.084 ± 0.003^†^	0.184 ± 0.029^†^	0.491 ± 0.081^†^
	SAITS	0.082 ± 0.008^†^	0.179 ± 0.054^†^	0.603 ± 0.080^†^
	**Ours**	**0.065 ± 0.006**	**0.130 ± 0.021**	**0.771 ± 0.062**
RCFT-Copy	GRU-M	0.125 ± 0.014^†^	0.570 ± 0.206^†^	0.353 ± 0.148^†^
	M-RNN	0.139 ± 0.013^†^	0.467 ± 0.109^†^	0.418 ± 0.159^†^
	SAITS	0.108 ± 0.006	0.377 ± 0.125	0.545 ± 0.099
	**Ours**	**0.100 ± 0.010**	**0.306 ± 0.063**	**0.631 ± 0.113**
SVLT- Delayed recall	GRU-M	0.128 ± 0.017^†^	0.575 ± 0.071^†^	0.354 ± 0.060^†^
	M-RNN	0.109 ± 0.001^†^	0.493 ± 0.059^†^	0.487 ± 0.105^†^
	SAITS	0.107 ± 0.011	0.439 ± 0.047	0.558 ± 0.066
	**Ours**	**0.096 ± 0.008**	**0.422 ± 0.031**	**0.626 ± 0.092**
RCFT- Delayed recall	GRU-M	0.113 ± 0.011^†^	0.897 ± 0.242^†^	0.351 ± 0.036^†^
	M-RNN	0.099 ± 0.003^†^	0.763 ± 0.178^†^	0.466 ± 0.032^†^
	SAITS	0.094 ± 0.008	0.758 ± 0.129	0.570 ± 0.059
	**Ours**	**0.085 ± 0.012**	**0.702 ± 0.143**	**0.634 ± 0.055**
COWAT	GRU-M	0.106 ± 0.006^†^	0.876 ± 0.896^†^	0.367 ± 0.054^†^
	M-RNN	0.095 ± 0.006	0.679 ± 0.671	0.495 ± 0.081
	SAITS	0.093 ± 0.011	0.684 ± 0.617	0.506 ± 0.078
	**Ours**	**0.086 ± 0.011**	**0.518 ± 0.354**	**0.601 ± 0.084**
Stroop color reading	GRU-M	0.153 ± 0.009^†^	0.672 ± 0.199^†^	0.426 ± 0.024^†^
	M-RNN	0.127 ± 0.009^†^	0.488 ± 0.171^†^	0.573 ± 0.126^†^
	SAITS	0.121 ± 0.009^†^	0.437 ± 0.131^†^	0.607 ± 0.103^†^
	**Ours**	**0.098 ± 0.007**	**0.365 ± 0.089**	**0.736 ± 0.070**
K-MMSE	GRU-M	0.084 ± 0.006^†^	0.886 ± 0.186^†^	0.211 ± 0.051^†^
	M-RNN	0.078 ± 0.011^†^	0.851 ± 0.160^†^	0.291 ± 0.052^†^
	SAITS	0.066 ± 0.006	0.700 ± 0.121	0.321 ± 0.098
	**Ours**	**0.066 ± 0.013**	**0.674 ± 0.185**	**0.418 ± 0.054**
CDR-SB	GRU-M	0.097 ± 0.011^†^	0.145 ± 0.043^†^	0.324 ± 0.051^†^
	M-RNN	0.090 ± 0.018^†^	0.126 ± 0.027^†^	0.377 ± 0.160^†^
	SAITS	0.069 ± 0.012	0.107 ± 0.022	0.537 ± 0.091
	**Ours**	**0.065 ± 0.009**	**0.097 ± 0.026**	**0.581 ± 0.088**

The values for the highest performance are highlighted in bold, while the second-highest performance is denoted with an underline. DSF, digital span forward; K-BNT, Korean version of the Boston Naming Test; RCFT, Rey complex figure test; SVLT, Seoul verbal learning test; COWAT, controlled oral word association test; K-MMSE, Korean-Mini Mental State Examination; CDR-SB, Clinical Dementia Rating Scale Sum of Boxes; MAE, mean absolute error; MAPE, mean absolute percentage error; GRU-M, mean imputation combined with gated recurrent unit; M-RNN, multi-directional recurrent neural network; SAITS, self-attention-based imputation for time series.

**Figure 2:**
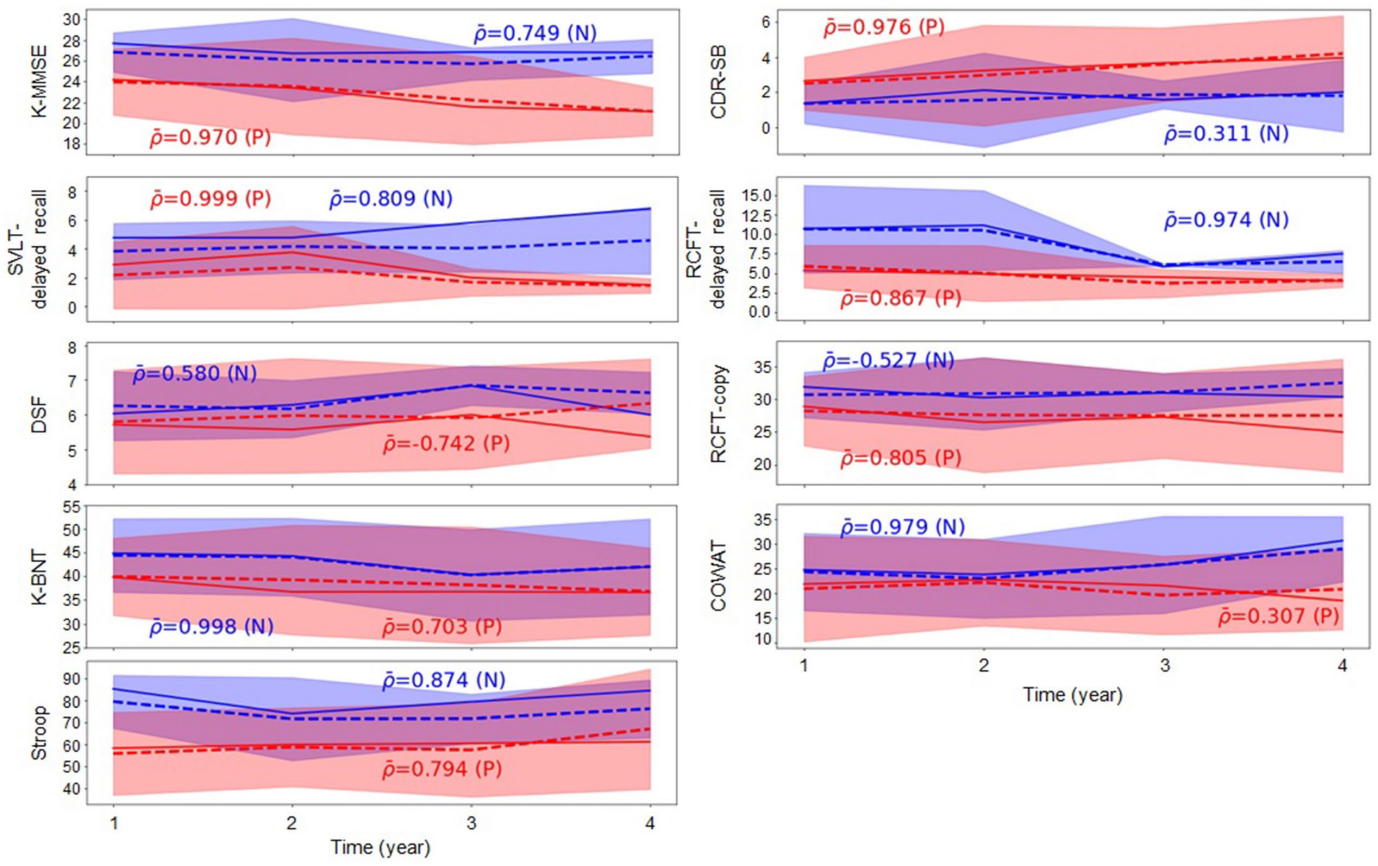
Group comparison of changes in the cognitive test scores over time. $\left(\bar{\rho }\right)$ represents the average Pearson correlation coefficient calculated within each group, where (*N*) and (*P*) denote groups with Aβ (−) and Aβ (+), respectively. Blue and red indicate the groups with Aβ (−) and Aβ (+), respectively. The solid line corresponds to the actual observations, whereas the dotted line depicts the predicted values. Aβ, amyloid-β; K-MMSE, Korean-Mini Mental State Examination; CDR-SB, Clinical Dementia Rating Scale Sum of Boxes; SVLT, Seoul verbal learning test; RCFT, Rey complex figure test; DSF, digital span forward; K-BNT, Korean version of the Boston Naming Test; COWAT, controlled oral word association test.

Table 2 indicates that, generally, all methodologies demonstrated high *R*^2^ values in the task of predicting cognitive test scores. Specifically, based on the *R*^2^ values resulting from our proposed method, the ranking of cognitive test scores was revealed as follows: K-BNT, Stroop Color Reading, RCFT-Delayed Recall, RCFT-Copy, SVLT-Delayed Recall, COWAT, CDR-SB, DSF, and K-MMSE. Furthermore, we conducted a group analysis of the groups with Aβ (+) and Aβ (−) aMCI to observe changes in the trajectory of the cognitive test scores over time and the intergroup differences in their values. Figure 2 presents the comparative analysis of the cognitive test scores between the groups with Aβ (+) aMCI and Aβ (−) aMCI. The findings indicate that the group with Aβ (+) exhibited a more rapid decline in cognitive tests, including the K-MMSE and CDR-SB, compared to the group with Aβ (−). When examining the trajectories of longitudinal cognitive scores in the group comparison, we observed that the cognitive scores of the Aβ (+) group tended to be lower than those of the Aβ (−) group, except for the CDR-SB score. However, higher values on CDR-SB indicate worse conditions; thus, this observation is meaningful. Based on this trend, the possibility of disease progression is expected to be higher in the group with Aβ (+) than that with Aβ (−) (Figure 2). Therefore, more attention should be focused on subjects with Aβ (+) MCI who are likely to exhibit rapid cognitive decline.

### Prediction of MRI markers

Similarly to Tables 2, 3 describes the prediction errors for longitudinal changes in MRI markers, assessed using three metrics: MAE, MAPE, and *R*^2^. Note that the values for the highest performance are highlighted in bold, while the second-highest performance is denoted with an underline. Just as with the cognitive test score predictions, our proposed method outperformed all considered competing methods, achieving the lowest MAE, MAPE, and *R*^2^ scores with statistical significance of p<0.05 for most competing methods. Furthermore, for this task, we observed a performance improvement in the order of GRU-M, M-RNN, SAITS, and our proposed method with the R^2^ values indicating much stronger performance in predicting MRI markers compared to the cognitive test score prediction task. Significantly, the MRI markers, including HV and cortical thicknesses in the occipital, cingulate, temporal, parietal, and frontal regions, yielded favorable predictive results, ranked in descending order. Several studies (Singh et al., 2006; Verfaillie et al., 2016; Lee et al., 2018) have reported consistent patterns of volume atrophy in various brain regions, particularly the prefrontal cortex, temporal lobe, and parietal lobe, in relation to cognitive decline. The extent of brain atrophy is predictive of cognitive decline over time.

**Table 3:** Performance of predicting MRI markers in terms of MAE, MAPE, and *R*^2^.

Features	Methods	MAE↓	MAPE↓	R^2^↑
Cingulate	GRU-M	0.080 ± 0.007^†^	0.082 ± 0.007^†^	0.514 ± 0.043^†^
	M-RNN	0.066 ± 0.008^†^	0.020 ± 0.004^†^	0.683 ± 0.109^†^
	SAITS	0.058 ± 0.007^†^	0.018 ± 0.002^†^	0.766 ± 0.102^†^
	**Ours**	**0.036 ± 0.006**	**0.011 ± 0.002**	**0.879 ± 0.047**
Frontal	GRU-M	0.060 ± 0.004^†^	0.060 ± 0.005^†^	0.555 ± 0.061^†^
	M-RNN	0.048 ± 0.008	0.016 ± 0.002	0.703 ± 0.086
	SAITS	0.048 ± 0.007	0.015 ± 0.002	0.709 ± 0.069
	**Ours**	**0.034 ± 0.003**	**0.011 ± 0.001**	**0.815 ± 0.032**
Parietal	GRU-M	0.066 ± 0.010^†^	0.070 ± 0.008^†^	0.543 ± 0.077^†^
	M-RNN	0.055 ± 0.008^†^	0.019 ± 0.002^†^	0.693 ± 0.028^†^
	SAITS	0.052 ± 0.006	0.018 ± 0.002	0.727 ± 0.104
	**Ours**	**0.038 ± 0.008**	**0.013 ± 0.002**	**0.822 ± 0.056**
Temporal	GRU-M	0.071 ± 0.011^†^	0.071 ± 0.013^†^	0.529 ± 0.083^†^
	M-RNN	0.056 ± 0.009^†^	0.017 ± 0.003^†^	0.693 ± 0.062^†^
	SAITS	0.052 ± 0.007	0.016 ± 0.003	0.746 ± 0.080
	**Ours**	**0.040 ± 0.002**	**0.013 ± 0.001**	**0.829 ± 0.040**
Occipital	GRU-M	0.079 ± 0.012^†^	0.078 ± 0.013^†^	0.557 ± 0.075^†^
	M-RNN	0.065 ± 0.008^†^	0.021 ± 0.002^†^	0.710 ± 0.084^†^
	SAITS	0.057 ± 0.004	0.019 ± 0.001	0.756 ± 0.068^†^
	**Ours**	**0.037 ± 0.006**	**0.013 ± 0.002**	**0.887 ± 0.043**
HV	GRU-M	0.022 ± 0.001^*^	0.096 ± 0.010	0.600 ± 0.058^†^
	M-RNN	0.015 ± 0.002^*^	0.085 ± 0.013	0.777 ± 0.093^†^
	SAITS	0.015 ± 0.001^*^	0.077 ± 0.009	0.809 ± 0.066^†^
	**Ours**	0.008 ± 0.002*	**0.049 ± 0.011**	**0.924 ± 0.023**

The values for the highest performance are highlighted in bold, while the second-highest performance is denoted with an underline. *: $\times 10^{-2}$ , †: p<0.05. HV, hippocampal volume; MAE, mean absolute error; MAPE, mean absolute percentage error; GRU-M, mean imputation combined with gated recurrent unit; M-RNN, multi-directional recurrent neural network; SAITS, self-attention-based imputation for time series.

**Figure 3:**
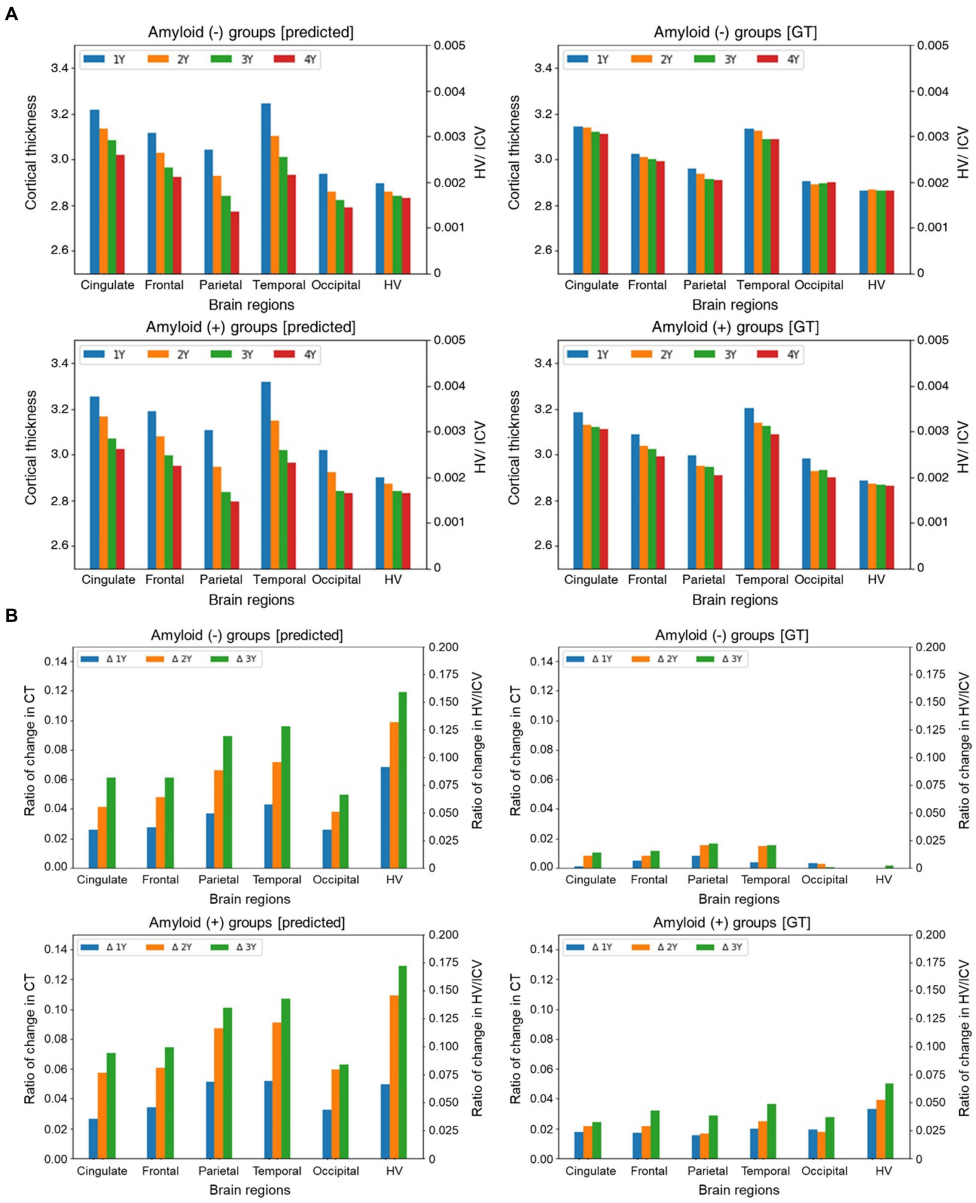
Group comparison of the trajectories for longitudinal MRI markers **(A)** and the ratio of relative changes in longitudinal MRI markers over time **(B)**. In figure **(A)**, the *x*- and *y*-axes represent different brain regions and cortical thickness and HV values are divided by ICV, respectively. In figure **(B)**, The *x*-axis represents different brain regions. The *y*-axis indicates the ratio of changes in cortical thickness and HV/ICV values relative to the baseline. The plots in each panel represent a specific time. Abbreviations: GT, ground truth; HV, hippocampal volume; ICV, intracranial volume; CT, cortical thickness.

Furthermore, we separately conducted a multifaceted analysis of the longitudinal cortical thinning in the groups with Aβ (+) and Aβ (−) to investigate the influence of the time interval from the baseline MRI scans. Initially, we examined the interaction effect between Aβ (+) and time, revealing that the group with Aβ (+) aMCI exhibited a significantly accelerated rate of cortical thinning than the group with Aβ (−) aMCI in specific brain regions, such as the cingulate, frontal, parietal, temporal, occipital, and hippocampal regions, as depicted in Figure 3A. Compared to the group with Aβ (−), those with Aβ (+) demonstrated a higher progression of brain region atrophy over time based on the ground truth (GT) in Figure 3A (right). Similarly, the predicted values also indicate that the group with Aβ (+) observed more significant brain region atrophy over time than the group with Aβ (−) in Figure 3A (left).

To delve deeper into the analysis, we further derived the following formula to quantify the relative changes in brain regions over time:


$$s_{g,t}^{\left(i\right)}=\frac{y_{t}^{\left(i\right)}-y_{0}^{\left(i\right)}}{y_{0}^{\left(i\right)}},s_{p,t}^{\left(i\right)}=\frac{{\hat{y}}_{t}^{\left(i\right)}-y_{0}^{\left(i\right)}}{y_{0}^{\left(i\right)}}\text{,}$$

where p,g,i, and t indicate the prediction, GT, indices of MRI markers, and time points, respectively. The calculated relative changes were subsequently normalized by the minimum and maximum values so that the resulting values were in the range of [−1,1], with positive and negative values representing increasing and decreasing, respectively. While there were some variations between the predictions and actual observations, we observed discernible differences between the groups. Specifically, the findings based on the GT demonstrated a greater progression of brain region atrophy in the group with Aβ (+) than that with Aβ (−). Regarding the predicted values, although a relatively minimal intergroup difference exists in brain region atrophy during the first year, starting from the second year, the group with Aβ (+) exhibits a more rapid progression of brain region atrophy than the group with Aβ (−) (Figure 3B).

**Figure 4:**
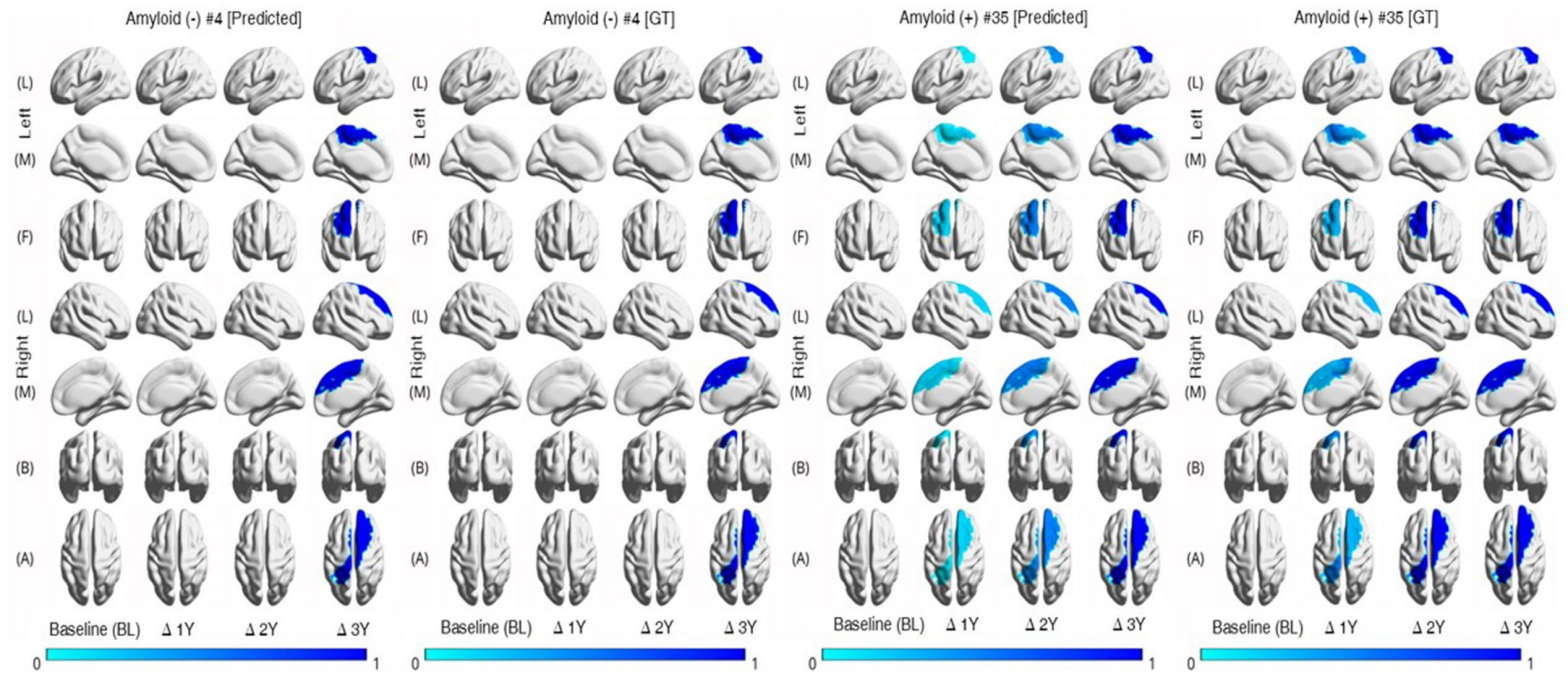
Individual trajectories of MRI markers representing relative changes over time. The color-coded values represent the normalized changes relative to the baseline values of the corresponding regions. The thickness of the brain region becomes thinner in Aβ (+) faster than Aβ (−). Aβ, amyloid-β; GT, ground truth.

Last, we conducted the individual-level changes in brain regions over time, as illustrated in Figure 4. In this study, we utilized the mean values for brain areas corresponding to each hemisphere. Consequently, as seen in Figure 4 [row (A)], there are overlapping areas when visualizing the results for specific regions. To enhance the clarity of these visualizations, we categorized the areas and separated them into left and right hemispheres. Following the calculations described in Figure 3B, we applied a threshold of 0.25 for the visualization process. The figure reveals that the degree of brain region atrophy is less prominent in the Aβ (−) samples until the second year. In contrast, the Aβ (+) samples exhibit substantial brain region atrophy from the first year onwards.

The group with Aβ (+) had a greater magnitude of cortical thinning and volumetric changes in specific brain regions over time than the group with Aβ (−) (Figures 3A,B). Further, similar patterns of analysis by group occurred, with variations in individual levels (Figure 4). Based on these findings, we concluded that the group with Aβ (+) is more likely to progress toward dementia or exhibit cognitive decline.

### Performance of the proposed framework (amyloid positivity prediction)

Although not the primary study focus, we conducted an analysis to indirectly assess the performance of the downstream task. We defined three scenarios for comparison: (1) comparing the diagnostic outcomes for first-time visiting patients and comparing the predictive results (2) with and (3) without missing values.

First, regarding the diagnostic outcomes of the first-time visiting patients, we employed the same input features as used in the proposed method, with the distinction that the time interval of observation was limited to 1 year. To evaluate the performance, we employed the support vector machine (SVM), which is widely used for classification tasks. We applied various metrics to assess the predictive performance, including accuracy, sensitivity, specificity, and the area under the receiver operating characteristic curve (AUC). The performance of this scenario is presented as follows: accuracy (0.731 ± 0.035), sensitivity (0.692 ± 0.057), specificity (0.765 ± 0.025), and AUC (0.779 ± 0.033).

The subsequent scenario encompassed predicting outcomes in the presence and absence of missing values. For the case of missing values (i.e., without applying an imputation task), the following procedure was employed to derive the results. As described in the experimental setting section, in the process of estimating missing values using the data-driven imputation approach, we evaluated the performance of the prediction of the MCI progression in patients by considering the imputation and classification loss values. The performance of longitudinal prediction for the case when values were missing is as follows: accuracy (0.706 ± 0.058), sensitivity (0.735 ± 0.088), specificity (0.735 ± 0.088), and AUC (0.779 ± 0.044). Based on these results, we replaced missing observations with imputed features at specific points in time.

When input observations were missing (i.e., applying an imputation task), missing observations were imputed through a previous step. Then, the final outputs (i.e., predicted MRI markers and cognitive scores) were estimated from the data in this study and the trained model. These final outputs were applied as input features and input into the SVM for the prediction task. The performance of longitudinal prediction when no values were missing is as follows: accuracy (0.756 ± 0.039), sensitivity (0.763 ± 0.046), specificity (0.819 ± 0.055), and AUC (0.814 ± 0.035). The results are higher than two cases, i.e., comparing the diagnostic outcomes for first-time visiting patients and the predictive results for longitudinal data with missing values, with a margin of 0.025 (vs. the first case) and 0.050 (vs. the second case) in accuracy, 0.039 (vs. the first case) and 0.028 (vs. the second case) in sensitivity, 0.054 (vs. the first case) and 0.084 (vs. the second case) in specificity, and 0.035 (vs. the first case) and 0.035 (vs. the second case) in AUC, respectively.

Based on these analytical findings, even with longitudinal data available, the presence of missing values could lead to similar or even lower classification performance than using single time-point data. However, using imputation methods to address missing values in longitudinal data, the results outperformed predictions based solely on single time-point data or longitudinal data with missing values. Theoretically, longitudinal data are expected to yield better performance due to the abundant information. However, the presence of missing values in longitudinal data can complicate the learning patterns of the models and sometimes result in inferior performance. One of the approaches for handling missing values is to remove them to alleviate this problem. However, in this study, we did not consider this approach because it could result in a loss of information inherent in the data. Consequently, we argue that longitudinal data yield better results than single time-point data; however, it is crucial to employ appropriate techniques for addressing missing values to mitigate their influence.

### Interpreting attention weights

**Figure 5:**
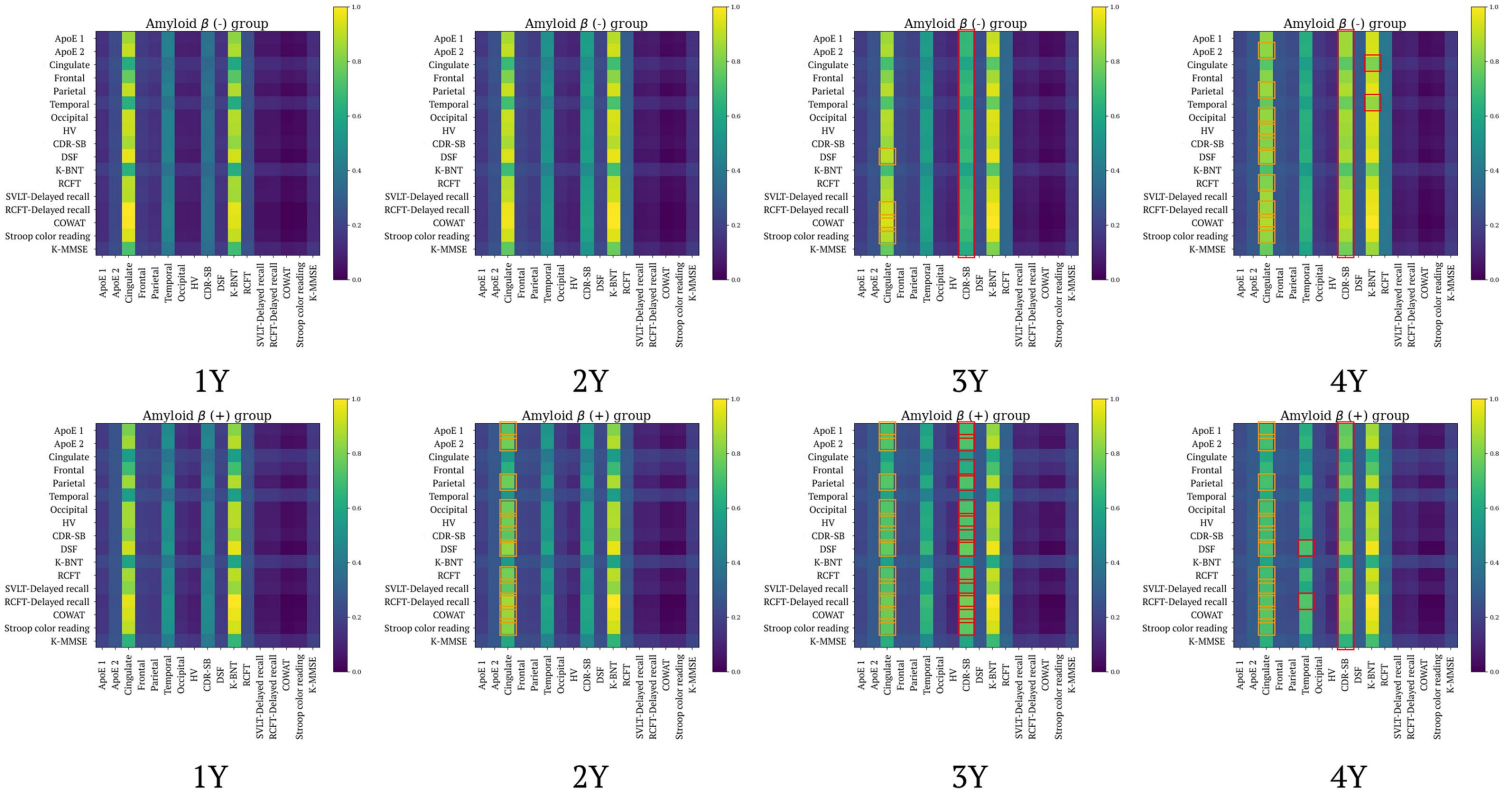
Group comparison of the weights of attention over time (**top**: amyloid negative group, **bottom**: amyloid positive group). Aβ, amyloid-β; ApoE, Apolipoprotein E (1: with ApoE ε4 allele, 2: without ApoE ε4 allele); CDR-SB, Clinical Dementia Rating Scale Sum of Boxes; COWAT, controlled oral word association test; dcCL, direct comparison Centiloid unit; DSF, digital span forward; HV, hippocampal volume; K-BNT, Korean version of the Boston Naming Test; K-MMSE, Korean-Mini Mental State Examination; RCFT, Rey complex figure test; SVLT, Seoul verbal learning test.

We aim to use the attention weights within our proposed model (e.g., the feature representation module-FRM) to indirectly interpret the basis for its performance in forecasting MRI biomarkers and clinical test scores, as well as amyloid positivity prediction. Specifically, we analyzed group-specific attention weights according to the patient’s clinical status at different time points to identify which input features contributed to identifying clinical status. At first, we observed that visualizing attention weights by group (e.g., classes) revealed not only the learning of different patterns across groups but also changes in these patterns over time (Figure 5). In Figure 5, attention weight values are derived from averaging across each group, then normalized to a range of [0,1] for each time sequence. The orange boxes indicate instances where the difference in attention weight values between the first observation and the respective time point exceeds 0.05, simultaneously suggesting a decrease in values over time. Conversely, the red boxes denote instances where the difference in attention weight values between the first observation and each subsequent time point is greater than 0.2, simultaneously indicating increased values over time. Through Figure 5, we observed the following findings: First, changes in Aβ (+) individuals begin to be noticeable from the 2-year (2Y), whereas in Aβ (−) individuals, no significant changes are observed at the same time sequence. Second, the observation of orange boxes for Aβ (−) indicates that as time progresses, differences in attention values emerge, leading to a pattern of attention changes in Aβ (−) that become similar to that observed in Aβ (+). However, the attention changes in Aβ (−) concerning *APOE* and SVLT features were not as marked as those in Aβ (+). Lastly, the most significant attention changes over time for both Aβ (+) and Aβ (−) were observed in the CDR-SB column. Specifically, for Aβ (+), significant attention changes were observed in DSF-Temporal and RCFT Delayed recall-Temporal, while for Aβ (−), significant changes were identified in Cingulate-K BNT and Temporal-K BNT.

## Discussion

The present study develops a predictive model using a deep recurrent network with an attention mechanism in carefully phenotyped and large cohorts who underwent molecular and structural imaging. The major findings are as follows. First, the proposed predictive model presents reliable predictions regarding cognitive decline and MRI markers over time. Second, faster cognitive decline and brain atrophy in larger regions were forecasted in patients with Aβ (+) than Aβ (−) MCI. The proposed method provides effective and accurate means for prognosis with individuals likely to progress within a specific period. By identifying subjects who are at a higher risk of rapid cognitive decline, the proposed model could accurately predict future cognitive decline and MRI marker changes over time for MCI patients at the individual level.

The prevalence of AD is expected to rise, with projections indicating that over 2% of the US population and 1% of the global population would be affected by AD by the year 2050 (Brookmeyer et al., 2007). Prior research has shown encouraging results in forecasting the timing of individuals’ progression to AD dementia using time-to-event analysis methods (Li et al., 2013, 2018; Barnes et al., 2014; Kong et al., 2015). Particularly, clinical and imaging-based measures at the baseline (Li et al., 2013; Barnes et al., 2014; Kong et al., 2015) and their longitudinal change trajectory (Li et al., 2018) have been adopted for predicting the progression of patients with MCI to AD dementia. Consistent with these studies, the findings also demonstrate that the amyloid PET data can offer valuable indicators for predicting the timing of patients’ development of AD from MCI.

Despite the importance of early detection and management of MCI and AD dementia for clinical practice and treatment, we still lack robust techniques for forecasting individual progression. To address this issue, we devise a simple but efficient deep learning-based prediction model that imputes missing observations and predicts cognitive outcomes using longitudinal data, drawing inspiration from Jung et al. (2021). However, our trajectory modeling approach differs from the previous work (Jung et al., 2021) in that it incorporates cognitive scores, brain imaging data, and amyloid PET data. Furthermore, our model could forecast the progression of MCI and identify subgroups with various patterns of progression according to Aβ positivity. Unlike conventional models that rely on AD spectrum-based diagnoses, our proposed model employs labels derived from amyloid PET scans. By integrating amyloid PET information, our model seeks to provide a more comprehensive and accurate representation of the pathological changes associated with AD, enhancing our prognostic models’ predictive power and specificity. Furthermore, our prognostic and predictive modeling targets the mid-point of the AD spectrum, specifically focusing on participants with (amnestic) MCI. Accordingly, the proposed method could provide a quantitative biomarker for predicting the longitudinal change of cognition and brain images simply by analyzing cognitive scores and brain images of Aβ (+) and Aβ (−) MCI patients. In the clinical setting, it would be more practical than simply predicting whether to proceed with AD (e.g., within a 3-year period of clinical assessment) because it would be possible to predict when each individual’s cognition may deteriorate by showing continuous information in detailed prognostic trajectories.

The proposed framework demonstrated superior performance across all evaluation metrics for the prediction task, outperforming the considered scenarios, including single time-point data and longitudinal data with missing observations. Furthermore, the proposed framework has the benefit of automatically forecasting MRI markers and reliable trajectories of the cognitive test scores. Notably, in the context of the cognitive scores, SVLT-Delayed recall, K-MMSE, and CDR-SB exhibited strong positive correlations, as indicated by the Pearson correlation coefficient. Similarly, in terms of MRI markers, the hippocampal, occipital, and cingulate regions demonstrated a significant correlation, as reflected by the coefficient of determination, making them the top three predictive variables for predicting the progression of MCI in patients.

Although our model generally shows high R^2^ value in predicting cognitive test scores after training on cognitive data, prediction efficacy is significantly increased when brain MRI data, including cortical thickness and hippocampal volume, is added. The proposed deep learning-based framework is capable of leveraging information from predefined anatomical regions with varying degrees of influence from the brain images while integrating cognitive scores. This capability facilitates accurate predictions and highlights the strengths of deep learning systems in using voxel-by-voxel levels in brain images. This is consistent with previous studies showing improved prediction of time to AD conversion when biological markers are included rather than neuropsychological data alone (Grueso and Viejo-Sobera, 2021; Franciotti et al., 2023). This could be explained by disease course, because Aβ biomarkers become abnormal first, followed by tau, followed by FDG PET and MRI based on biomarker model of pure AD (Jack et al., 2013). Cognitive impairment is the last event in the progression of the disease (Jack et al., 2013). These results highlight the importance of utility of brain images in predicting the course managing MCI and AD patients at different stages of the disease process.

The strengths of this study are the standardized imaging procedures and thorough clinical evaluation. Using this proposed method, clinicians can identify patients at risk for cognitive decline and offer timely intervention to reduce risk, provide potential treatment, and assist patients and families in planning. Recently, anti-amyloid monoclonal antibodies have shown some promise for slowing cognitive decline and adverse brain structure changes in MCI and early AD (van Dyck et al., 2022; Cummings, 2023; Sims et al., 2023). Our method allows clinicians to identify patients at risk of worsening using our proposed method in outpatient clinics in advance and apply these medications to delay the onset of dementia or even prevent disease. However, this study has several limitations. First, the robustness of the proposed framework is restricted by the clinical distribution of the SMC training dataset. Furthermore, this SMC training set excluded cases of non-AD neurodegenerative disorders, restricting the applicability of the algorithm to this patient population. We used Aβ PET uptakes and cortical thickness measurements from MRI to measure Aβ neurodegeneration, due to the lack of available pathological confirmation. However, we were unable to account for other pathologies contributing to neurodegeneration, such as tau, transactive response DNA-binding protein (TDP-43), hippocampal sclerosis, and argyrophilic grain disease. As a result, our findings may be limited in generalizability. Therefore, external validation using other datasets from different cohorts should be conducted in the future to further assess the model’s generalizability and robustness across diverse populations. Second, the diagnosis of MCI is inherently unstable because its accuracy relies on the duration of follow-up. For instance, some patients with MCI may have eventually developed AD if they had been followed for a sufficient time. Moreover, in the present study, neuropsychological tests were performed using SNSB-II, which may not be directly comparable to the test batteries more commonly used in international research contexts. However, all of the included tests in SNSB have been internationally used for several decades in clinical practice. The items used in the tests were altered due to the linguistic and cultural differences between Korean and English speakers. Therefore, the model’s predictions would remain valid if applied to populations assessed with different neuropsychological instruments, but further research is needed.

However, this study demonstrates that the proposed framework could forecast cognitive scores and imaging markers with high accuracy. By calibrating the model and conducting extensive external validation using data from many institutions, the algorithm could be included into the clinical workflow and function as a crucial decision-support tool, assisting clinicians in the early prediction of MCI progression.

## Conclusion

In conclusion, the proposed framework for predicting the progression of MCI in patients could demonstrate promising performance, assisting in differentiating subjects with MCI with different progression patterns. This method also identifies subjects with MCI with a higher progression risk, providing cost-effective treatment at the individual level. Furthermore, this framework has the ability to facilitate the process of enrolling patients in clinical trials who are likely to progress within a specific time frame. We anticipate that this approach can aid in selecting patients with MCI who would benefit from early intervention.

## Data availability statement

The raw data supporting the conclusions of this article will be made available by the authors, without undue reservation.

## Ethics statement

The studies involving humans were approved by Institutional Review Board of Samsung Medical Center (IRB No: 2018–10-120). The studies were conducted in accordance with the local legislation and institutional requirements. The participants provided their written informed consent to participate in this study.

## Author contributions

WJ: Conceptualization, Formal analysis, Methodology, Resources, Writing – original draft, Writing – review & editing. SK: Conceptualization, Methodology, Writing – original draft, Writing – review & editing. JK: Resources, Writing – review & editing. HJ: Resources, Writing – review & editing. CP: Resources, Writing – review & editing. HK: Resources, Writing – review & editing. DN: Resources, Writing – review & editing. SS: Conceptualization, Funding acquisition, Methodology, Resources, Supervision, Writing – original draft, Writing – review & editing. H-IS: Conceptualization, Formal analysis, Funding acquisition, Methodology, Resources, Supervision, Writing – original draft, Writing – review & editing.

## Footnotes

^1^http://www.gaain.org
